# Nested PCR for mtDNA-4977-bp deletion and comet assay for DNA damage - a combined method for radiosensitivity evaluation of tumor cells

**DOI:** 10.3892/ol.2014.1819

**Published:** 2014-01-22

**Authors:** JIANGUO LI, YAN WANG, LIQING DU, CHANG XU, JIA CAO, QIN WANG, QIANG LIU, FEIYUE FAN

**Affiliations:** 1 Institute of Radiation Medicine, Chinese Academy of Medical Sciences and Peking Union Medical College, Tianjin Key Laboratory of Molecular Nuclear Medicine, Tianjin 300192, P.R. China; 2 Department of Human Anatomy, The Medical School of Inner Mongolia University for the Nationalities, Tongliao, Neimenggu 028041, P.R. China

**Keywords:** radiosensitivity, mitochondrial DNA, comet assay, nested PCR, DNA damage

## Abstract

To identify an effective method of evaluating the radiosensitivity of human tumor cell lines *in vitro*, the present study adopted mtDNA-4977-bp deletion coupled with comet assay. The three human tumor cell lines applied were HepG_2_, EC-9706 and MCF-7. The surviving fraction (SF), ratio of the mtDNA-4977-bp deletion and DNA damage were detected by MTT assay, nested polymerase chain reaction (PCR) technique and comet assay, respectively. Clearly, lower SFs were found for the HepG_2_ and EC-9706 cells as compared with the MCF-7 cells following irradiation at doses of 2, 4 and 8 Gy, indicating a higher radiosensitivity for the HepG_2_ and EC-9706 cells. Additionally, no significant differences were identified in the mtDNA-4977-bp deletions found among HepG_2_, EC-9706 and MCF-7 cells by PCR following 1- or 4-Gy γ-ray irradiation, while increased deletion ratios of mtDNA-4977 bp were observed in HepG_2_ and EC-9706 cells following 8-Gy irradiation, in contrast to decreases in MCF-7 cells. The most notable differences among these three tumor cell lines were observed by comet assay following 8–Gy γ-ray irradiation. A combined method of nested PCR and comet assay, therefore, is the most effective and accurate method in evaluating the radiosensitivity of tumor cells.

## Introduction

Radiotherapy is currently one of the most important tumor treatments. Due to the different radiosensitivities of tumors in different individuals, even tumors sharing the same tissue type and occurring in the same organ (1), an effective therapy based on the radiosensitivity of an individual patient may be improved through a better prediction of the radiosensitivity of the patient’s primary cell cultures (2). A number of biological markers have been adopted for predicting the radiosensitivity of tumor cells, but have been imperfect in their lack of specificity. The deletion of mtDNA, a new radiobiological endpoint (3,4), has recently been directly detected, providing a fast method of predicting the radiosensitivity of tumor cells. There are 100 to 1,000 mtDNA molecules in a single cell. As a unique extranuclear hereditary substance located in the mitochondrial inner membrane, mtDNA presents a mutation rate far higher than nuclear DNA (10 to 20 times) and has become a hot spot in mutation studies for its vulnerability to active oxyradical damage and its lack of an effective restoration system and the protection of histones (5).

Double- or single-strand breaks occur in tumor cells following irradiation and the DNA breaks in a cell are intimately associated with its radiosensitivity (2,4,6–9). A number of methods have been identified for assaying DNA damage, including DNA filter elution, constant field gel electrophoresis, pulsed-field gel electrophoresis and comet assay (2,10,11). Comet assay, also called single cell gel electrophoresis (SCGE), is considered to be a sensitive, fast and convenient technique for detecting DNA damage and repair in a single cell (12,13) and it is, therefore, a prospective radiosensitivity prediction method for clinical use (2).

In the present study, three human tumor cell lines, HepG_2_, EC-9706 and MCF-7, were applied. The radiosensitivity of the tumor cells was evaluated by three different methods, namely, MTT assay, nested polymerase chain reaction (PCR) and comet assay. The results of the three methods were combined and compared for the comprehensive analysis of the multiple biological parameters (MTT, ΔmtDNA-4977 and comet assay) affecting the radiosensitivity prediction of tumor cells.

## Materials and methods

### Cell culture and MTT

The tumor cell lines, HepG_2_, EC-9706 and MCF-7, purchased from the Cell Culture Centre of the Chinese Academy of Medical Science nad Peking Union Medical College (Beijing, China) and were conserved by the Radiation Hazard Evaluation Laboratory of the Chinese Academy of Medical Science and Peking Union Medical College (Tianjin, China). Cells were maintained in RPMI-1640 as suspensions at densities of 1×10^5^/ml and then irradiated by a ^137^Cs γ-ray with a dose rate of 1.23 Gy/min and dose points of 0, 1, 2, 4 and 8 Gy. Following inoculation in 96-well plates with 200 μl of each irradiation dose (six copies) per well, cells of the irradiated group and a non-irradiated group as control were cultured at 37°C in humidified conditions at 5% CO_2_ for three days. Then, 20 μl MTT at a concentration of 5 mg/ml was added to each well and co-incubated for 4 h. Following the removal of raffinate, 150 μl dimethyl sulfoxide was added to every well and the absorption value (A) was evaluated by an ELISA reader (Thermo Electron Corporation, Waltham, MA, USA) at a wavelength of 492 nm. The surviving fraction (SF) was calculated using the following formula: $\text{SF}={\text{A}}_{\text{irradiated group}}/{\text{A}}_{\text{non}-\text{irradiated group}}$.

### Nested PCR

Cells with a density of 5×10^7^/ml were irradiated by a ^137^Cs γ-ray with a dose rate of 1.23 Gy/min and dose points of 0, 1, 4 and 8 Gy prior to incubation at 37°C for 2 h. Subsequently, mtDNA was extracted according to the manufacturer’s instructions (Shanghai Genmed Gene Pharmaceutical Technology Co., Ltd., Shanghai, China). As shown in Table I, primers P1–P2 were used in the amplification of the internal standard, which was considered as the total mtDNA with a segment of 533 bp and three pairs of nested primers (P3–P4, P5–P6 and P7–P8) were utilized to amplify the deletion of mtDNA-4977 bp (3).

The reaction conditions for P1–P2 were as follows: Initiation at 94°C for 5 min, 55°C for 5 min and 72°C for 3 min; followed by an amplification of 34 cycles at 94°C for 40 sec, 55°C for 40 sec and 72°C for 50 sec. The PCR of P3–P4 was performed for 35 cycles, including 3 min of pre-denaturation at 94°C, then amplification at 94°C for 1 min, 45°C for 1 min and 72°C for 1 min. No PCR product was obtained when the deletion of the mtDNA occurred following irradiation for an insufficient time, for the extension of a segment longer than 5 kbs in normal mtDNA when the reaction condition was tightly controlled.

Products were separated through 1% agarose gel and stained with ethidium bromide (0.5 μg/ml). The intensity of each band was collected by a Gel Doc 100 imaging system (United Scientific Supplies, Inc., Waukegan, IL, USA) and analyzed by Molecular Analysis™ software (Biocompare, South San Francisco, CA, USA) for band quantification. The deletion rate of mtDNA-4977 bp was confirmed by ratios of the band intensity of the mtDNA-4977-bp deletion compared with that of the internal standard.

### Comet assay

An alkaline comet assay was performed according to Qiang *et al* (11) and Banáth *et al* (14). A 25-μl cell suspension at a concentration of 2×10^5^/ml was mixed with 75 μl low-melting agarose (0.75%; solidified at 4°C; BIOWEST, Barcelona, Spain) and the mixture was layered onto a slide that had been covered with 100 μl normal-melting agarose (0.75%; solidified at 4°C; BIOWEST). The slides were placed immediately into a lysing solution at 4°C for 2 h and then washed three times. The slides were allowed to rest for 20 min and then electrophoresis was conducted for 20 min at 20 V and ~200 mA. The slides were stained with ethidium bromide (2 μg/ml) and washed with double-distilled water. Finally, 100 comet images were captured for every sample using a fluorescence microscope (Nikon corporation, Tokyo, Japan). The computer automated stowage planning (CASP) automatic analysis system provided by Wrocław University (Wrocław, Poland) was utilized to analyze the comet images (15) and the tail moment (TM) and olive tail moment (OTM) induced by irradiation were considered as the analysis parameters.

### Statistical analysis

The experimental data used in the statistical analysis are presented as the mean ± standard deviation, since six parallel copies of each sample were used. The SF, deletion ratio of mtDNA-4977 bp and TM and OTM of the comet were examined using one-way analysis of variance. All statistical analyses were performed using the SPSS 13.0 (SPSS, Inc., Chicago, IL, USA) statistical software for Windows. P<0.05 was considered to indicate a statistically significant difference.

## Results

### SF of tumor cells following γ-ray irradiation

The SFs of the tumor cells following γ-ray irradiation at various doses are shown in Table II. At the same dose, HepG_2_ cells showed the lowest SF and MCF-7 the highest among the three different cell lines. Manifest differences were observed in the SF of MCF-7 cells compared with HepG_2_ (P<0.05) or EC-9706 (P<0.01) cells following irradiation at doses of 2, 4 and 8 Gy. This observation markedly demonstrates the higher radiosensitivities of HepG_2_ and EC-9706 cells. Additionally, no statistical difference was identified between the SFs of the HepG_2_ and EC-9706 cells (P>0.05).

### Loss of mtDNA-4977 bp following γ-ray irradiation

The PCR product of P1–P2 as the internal standard was 533 bp long, coinciding with the design of the current study. In accordance with our prediction, the end product of the nested PCR was 391 bp long following irradiation covering the deleted section of 4,977 bp, while it was not found in cells without irradiation. Alongside an increase in the irradiation dose, an increase of the mtDNA-4977-bp deletion was observed (Table III). No significant difference was identified in the deletion ratios of mtDNA-4977 bp between 1 and 4 Gy (P>0.05). However, increased deletion ratios of mtDNA-4977 bp were observed in HepG_2_ and EC-9706 cells following 8-Gy irradiation, with contrasting decreases in MCF-7 cells. Due to the statistical differences between HepG_2_ and MCF-7, as well as EC-9706 and MCF-7 (P<0.05), it was concluded that the radiosensitivities of HepG2 and EC-9706 cells were higher than those of MCF-7 cells. No significant difference was identified between HepG_2_ and EC-9706 cells (P>0.05).

### Comet assay

**Figure 1:**
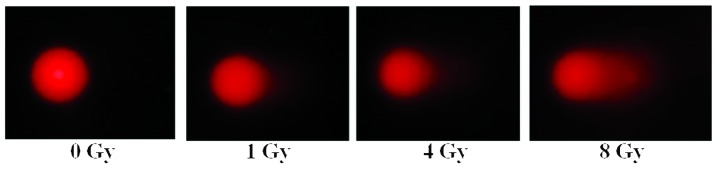
Comet images of HepG_2_ cells following irradiation at various doses.

**Figure 2:**
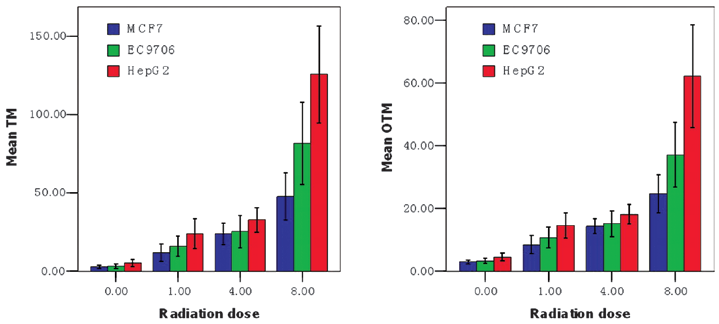
Comparison of the TM and OTM of three tumor cells following irradiation at various doses. TM, tail moment; OTM, olive tail moment.

Irradiation with the 0- to 8-Gy γ-ray led to the breakage of DNA chains. Following unwinding, DNA fragments left the nuclear zone and moved to the positive pole under the effect of the electric field in the electrophoresis liquid, forming the distinctive comet tail formation (Fig. 1). A manifest difference of radiosensitivity was observed in three tumor cell types following irradiation at 8 Gy, as shown in Fig. 2. Significant differences were found for all the groups, with the exception of the comparison between MCF-7 and EC-9706 cells at the dose of 4 Gy (Table IV).

## Discussion

The deletion of mtDNA-4977 bp is in the locus between a 13-bp long direct repeat sequence in the mtDNA and is the most common mutation caused by radiation (16,17). The application of nested PCR to amplify the mtDNA-4977-bp deletion induced by irradiation markedly overcomes the instability of ordinary PCR and accordingly ensures the specificity of the result (18). The current study confirmed that the mtDNA-4977-bp deletion occurs in all the tumor cells following irradiation at any dose, suggesting that the mtDNA-4977-bp deletion is one of the markers of radiation damage. Previously, Kubota *et al* (3) presumed that the radiosensitivity of cells may be evaluated by the radiation dose capable of inducing the deletion of mtDNA-4977 bp. The present study performed a quantitative analysis on the mtDNA-4977-bp deletions induced by a certain dose of irradiation (as opposed to the inability to quantitatively predict radiosensitivity in the study by Kubota *et al*) and, thereby, allows for the evaluation of the radiosensitivity of different cells.

Significant differences were observed between radiation-sensitive and -insensitive tumor cells following γ-ray irradiation, as observed using the comet assay, which demonstrated that the degree of DNA damage caused by irradiation may reflect the radiosensitivity of cells (2). This strong association between DNA damage and radiosensitivity has also been observed by other studies (2,19,20), while certain studies contradict this point of view due to their investigation of the double-strand breakage of DNA (10,21). The present study detected the single-strand breakage of DNA by the SCGE method and a manifest difference was found among the three types of tumor cells with different radiosensitivities at the highest dose of 8 Gy. Therefore, the comet assay reflects the radiosensitivities of tumor cells at high doses of irradiation.

SF_2_ (irradiated at a dose of 2 Gy) calculated by MTT has been considered to be a reliable approach for evaluating radiosensitivity, but it is limited in clinical use by its toilsome and time-consuming cell culture demands (22). The present study used this method as a control, wherein lower values of SF_2_ determined higher radiosensitivities. The SF_2_ values for the HepG_2_, EC-9706 and MCF-7 cells were 0.53, 0.58 and 0.79, respectively, which markedly indicated the lowest radiosensitivity for MCF-7. Similarly, the radiosensitivities of HepG_2_ and EC-9706 were also higher than those of MCF-7 following irradiation at 4 and 8 Gy. Investigation of the mtDNA-4977-bp deletion was unable to determine the radiosensitivity of tumor cells following irradiation at 1 and 4 Gy, but higher radiosensitivities of HepG2 and EC-9706 compared with MCF-7 were found following irradiation at 8 Gy, consistent with the results of the MTT. Additionally, the two methods showed no difference in radiosensitivity between HepG_2_ and EC-9706 cells. As found in SCGE, a manifest difference was identified among the three types of tumor cells with various radiosensitivities at the highest dose of 8 Gy. In brief, selecting two or more biological markers for simultaneous determination results in a more accurate prediction of the radiosensitivity of tumor cells. However, this strategy remains to be better defined and future studies must be performed to determine the optimal dose, maximal efficiency, accuracy and practicability.

## Figures and Tables

**Table I:** Primers of mtDNA used for nested polymerase chain reaction.

Segments	Primers	Position	Length, bp
Internal standard			
P1	5′-aacatacccatggccaacct-3′	3304–3836	533
P2	5′-ggcaggagtaatcagaggtg-3′		
First cycle			
P3	5′-tgaacctacgagtacaccga-3′	7901–14220	1342
P4	5′-ttagtagtagttactggttg-3′		
Second cycle			
P5	5′-ttcatgcccatcgtcctaga-3′	8201–13851	673
P6	5′-gttgaggtctagggctgtta-3′		
Third cycle			
P7	5′-cccctctagagcccactgtaaagc-3′	8282–13650	391
P8	5′-ggggaagcgaggttgacctg-3′		

**Table II:** Surviving fraction values of tumor cells following γ-ray irradiation (mean ± standard deviation).

Cells	1 Gy	2 Gy	4 Gy	8 Gy
HepG_2_	0.89±0.17	0.53±0.10^a^	0.27±0.06^b^	0.09±0.04^b^
EC-9706	0.91±0.18	0.58±0.11^a^	0.34±0.06^b^	0.13±0.03^b^
MCF-7	0.93±0.18	0.79±0.13	0.52±0.08	0.24±0.06

a P<0.05 and

b P<0.01, vs. MCF-7 (n=6).

**Table III:** Deletion ratio of mtDNA-4977 bp following γ-ray irradiation.

Cells	0 Gy	1 Gy	4 Gy	8 Gy
HepG_2_	0	0.26±0.06	0.31±0.06	0.43±0.09^a^
EC-9706	0	0.30±0.06	0.34±0.05	0.39±0.07^a^
MCF-7	0	0.35±0.07	0.37±0.08	0.27±0.05

a P<0.05, vs. MCF-7 (n=6).

**Table IV:** P-values for comparing the TM and OTM of three tumor cells by comet assay.

		EC9706		HepG_2_	
			
Dose, Gy	Cells	TM	OTM	TM	OTM
0	MCF-7	0.037	0.011	0.000	0.000
	EC9706			0.000	0.000
1	MCF-7	0.000	0.000	0.000	0.000
	EC9706			0.000	0.000
4	MCF-7	0.271	0.122	0.000	0.000
	EC9706			0.000	0.000
8	MCF-7	0.000	0.000	0.000	0.000
	EC9706			0.000	0.000

TM, tail moment; OTM, olive tail moment.
